# ccfDNA analysis for the classification of adrenocortical adenomas

**DOI:** 10.1007/s40618-025-02540-5

**Published:** 2025-02-01

**Authors:** Mengjie Xu, David S. Tourigny, Juliane Lippert, Ana Crastin, Silke Appenzeller, Miriam Asia, Oskar Podstawka, Gabrielle Smith, Yasir S. Elhassan, Kassiani Skordilis, Alessandro Prete, Cristina L. Ronchi

**Affiliations:** 1 Department of Metabolism and Systems Science, College of Medicine and Health, University of Birmingham, Birmingham, UK; 2 Department of Endocrinology, Taihe Hospital, Hubei University of Medicine, Shiyan, P. R. China; 3 School of Mathematics, University of Birmingham, Birmingham, UK; 4 Institute of Human Genetics, University of Wuerzburg, Wuerzburg, Germany; 5 Core Unit Bioinformatics, Comprehensive Cancer Center Mainfranken, University Hospital Wuerzburg, Wuerzburg, Germany; 6 Department of Endocrinology, Queen Elizabeth Hospital Birmingham NHS Trust, Birmingham, UK; 7 Department of Cellular Pathology, Queen Elizabeth Hospital Birmingham, Birmingham, UK; 8 Birmingham Biomedical Research Centre, NIHR, University of Birmingham and University Hospitals Birmingham NHS Foundation Trust, Birmingham, UK

**Keywords:** Adrenocortical tumours, Circulating cell-free DNA, Cortisol excess, Primary aldosteronism, Next-generation sequencing, Liquid biopsy

## Abstract

**Background:**

Somatic alterations are commonly observed in adrenocortical adenomas including cortisol-producing (CPA) [overt Cushing syndrome (CS) or mild autonomous cortisol secretion (MACS)], aldosterone-producing (APA), and non-functioning (NFAT) tumors. We tested whether somatic variants could be detected in circulating cell-free DNA (ccfDNA) from patients with adenomas and potentially contribute to management strategies.

**Materials and methods:**

We investigated 44 patients (17 CPA-MACS, 9 CPA-CS, 12 APA, and 6 NFAT). 23 healthy subjects (HS) served as controls. ccfDNA was extracted from blood samples and quantified with fluorimeter. Tumor DNA (T-DNA) was isolated from paraffin embedded tissue in 17/44 cases. Matched ccfDNA/T-DNA were sequenced using a customized panel including 32 genes. Leucocyte DNA was used to filter out germline variants.

**Results:**

Patients with adenomas had higher total ccfDNA concentrations than HS [median 0.12 (IQR 0.05–0.19) vs. 0.05 (0.00-0.08) ng/µl, *P* < 0.001], with CPA-CS showing the highest ccfDNA levels [0.18 (0.05–0.47) ng/µl]. Within T-DNA, somatic variants were identified in 53% of adenomas: *PRKACA* in 2/7 CPA-CS, *CTNNB1* in 3/5 CPA-MACS and 1/7 CPA-CS, *KCNJ5* in 2/5 APA and *CACNA1D* in 1/5 APA. Somatic mutations were not detected in any of the investigated ccfDNA samples.

**Conclusions:**

Total ccfDNA concentrations are higher in patients with CPA-CS. Despite the presence of somatic variants in half of tumor samples, we did not detect any at ccfDNA level. Therefore, this approach appears ineffective for pre-operative detection of genetic alterations.

**Supplementary Information:**

The online version contains supplementary material available at 10.1007/s40618-025-02540-5.

## Introduction

Adrenocortical tumours, most of which represent benign adrenocortical adenomas (ACA), are present in 3–7% of adults and are diagnosed incidentally in the vast majority of cases [1]. ACA are mostly non-functioning (NFAT) but can be cortisol-producing (CPA), with overt Cushing’s syndrome (CS) or mild autonomous cortisol secretion (MACS), or aldosterone-producing (APA). MACS is the most frequent hormone abnormality in ACA (20–50% of cases) [1], and chronic exposure to cortisol excess is associated with increased cardiometabolic burden including central obesity, diabetes mellitus, hypertension, osteoporosis, and increased mortality risk [2–4]. As a result, the 2023 ESE-ENSAT guideline on adrenal incidentalomas recommends that patients with ACA are systematically assessed for MACS to guide management and follow-up [1]. Primary aldosteronism is the most common cause of secondary hypertension [5] and is associated with an increased risk of cardiovascular and cerebrovascular events [6]. Primary aldosteronism exists on a spectrum ranging from unilateral APA, which can be successfully treated by adrenalectomy, to bilateral adrenocortical hyperplasia (BAH), which is managed with mineralocorticoid receptor antagonists [7]. Distinguishing APA from BAH currently relies on adrenal venous sampling (AVS), an invasive procedure which is not widely available [8].

Alterations of components of the cAMP/PKA pathway, including somatic variants in *PRKACA* (encoding for the catalytic subunit of the protein kinase A) and *GNAS* (encoding for the stimulatory G protein alpha-subunit), have been identified as causative factors for up to 50–60% of CPA associated with CS (CPA-CS) [8, 9]. Activating variants of *CTNNB1* (encoding for beta-catenin) have been observed in 20–60% of CPA-MACS and NFAT [9, 10] and rarely in APA (~ 3–5%). In unilateral APA, the most frequent somatic variants are found in *KCNJ5* (encoding for the potassium inwardly-rectifying channel, subfamily J, member 5), involved in the calcium/calmodulin kinase signalling and mutated in ~ 40% of cases [11]. Patients harbouring *KCNJ5* mutations have better clinical outcomes after surgery, while *CTNNB1* mutations are associated with a higher likelihood of residual hypertension after adrenalectomy [6, 12].

Dissecting the molecular landscape of ACA has improved our understanding of disease classification and prognostication [13], but this typically relies on analysis of the tumour tissue, only available for patients undergoing adrenalectomy. Circulating cell-free DNA (ccfDNA) can contain DNA released by tumour cells and therefore serve as a surrogate for DNA extracted directly from the tumour [14]. Somatic mutations can be detected in ccfDNA of patients with different solid cancer types [15], including adrenocortical carcinoma [16]. Although studies on ccfDNA in benign tumors are limited, existing literature suggests that ccfDNA levels are generally lower in benign compared to malignant tumours (e.g. in breast, thyroid, colon). Moreover, genetic alterations at ccfDNA level are generally less frequent in benign tumours and cancer-specific mutations present in malignant nodules are absent in benign counterparts, potentially useful for differential diagnosis [17–19].

However, the possibility of detecting somatic mutations in the ccfDNA of patients with benign adrenal tumours remains unexplored.

In the present study, we hypothesized that detecting somatic variants in blood samples pre-operatively could guide the personalized management of patients with ACA. We aimed to establish whether somatic variants within genes commonly mutated in adrenal tumours can be detected before surgery in ccfDNA from patients with ACA. We also investigated the relationship between ccfDNA-based biomarkers and the clinical phenotype.

## Materials and methods

### Patient cohort

We recruited adult patients with newly diagnosed ACA referred to the Adrenal Tumour Service at the Queen Elizabeth Hospital Birmingham between August 2018 and July 2023. ACA was diagnosed based on benign radiological characteristics according to current guidelines [1]. Patients with conditions that could significantly affect circulating cortisol levels, such as severe chronic inflammatory or autoimmune diseases, severe liver or kidney failure, and treatment with oral glucocorticoids or other immunomodulatory drugs were excluded. Adrenal hormone excess was defined as per the 2023 ESE-ENSAT guidelines [1]. CPA-CS was defined by the presence of clinical features of CS together with suppressed plasma adrenocorticotropic hormone (ACTH) levels and at least two positive screening tests for hypercortisolism (overnight 1 mg-dexamethasone suppression test (ONDST), late-night salivary cortisol, or 24-h urine free cortisol). MACS was defined by the failure to suppress cortisol after the ONDST in the absence of overt clinical features of CS. Diagnosis of APA was based on at least two paired renin and aldosterone results (aldosterone-renin-ratio, ARR) and a saline infusion test when required [20]. AVS was performed to lateralise the source of aldosterone excess as per current guidelines [1, 20]. Patients with normal ONDST and ARR results were classified as having NFAT.

Demographics, hormonal data (i.e. cortisol levels after ONDST, serum dehydroepiandrosterone sulphate (DHEAS), aldosterone levels, and plasma ACTH and renin levels) and radiological data were collected from medical records. In case of multiple adenomas, the diameter of the largest mass was reported.

The cases of patients with clinically-relevant steroid excess were discussed in our multi-disciplinary team meetings and, when recommended, surgical removal of ACA by minimally-invasive adrenalectomy was performed in our hospital by expert surgeons.

Blood samples were also collected from 23, as far as known, healthy subjects (HS) recruited among university staff that served as controls for the baseline ccfDNA concentration analysis, as previously published [16].

This study has been conducted in accordance with the Declaration of Helsinki. Institutional ethical approval was obtained (PrimeAct REC 20/NW/0207). All participants provided informed written consent.

## Blood sample collection and processing

We have previously established a standardised pipeline for sample collection and processing to obtain reliable ccfDNA samples [16]. In brief, 10–20 ml of blood was collected in EDTA tubes and kept on ice until centrifuged (within 2–3 h of blood collection) for 10 min at 15 °C and 2400 rpm. After centrifugation, plasma was transferred to clean centrifugation tubes without disturbing the buffy coat and centrifuged for another 10 min at room temperature and 13,000 rpm. Plasma was then transferred to a fresh centrifugation tube without disturbing the pellet and stored at −80ºC until analysis. Germline DNA was isolated from matched peripheral blood samples using the NucleoSpin Blood L Kit (Macherey-Nagel, Bethlehem, PA, USA) according to the manufacturer’s instructions.

## ccfDNA quantification and sequencing

### ccfDNA isolation

ccfDNA was isolated from 2 to 6 ml of plasma with the Cell3™ Xtract kit (Nonacus, Birmingham, UK) according to manufacturers’ instructions.

### ccfDNA concentration measurement and quality control

ccfDNA concentrations were determined by Quantus™ Fluorometer (Promega, Fitchburg, United States) according to the manufacturer’s instructions. Different volumes of plasma taken for ccfDNA isolation were considered for the designation of the final ccfDNA concentration in a sample. A quality control (QC) for the desired fragment length of the ccfDNA (150–250 bp) was performed on a TapeStation High Sensitivity 1000D system and the TapeStation Analysis Software 4.1.1 (Agilent, Santa Clara, United States). All ccfDNA samples included in the analysis showed good quality in means of fragment length and no contamination levels with high molecular weight material. Representative examples of QC by TapeStation in both ACA and HS samples are shown in **Suppl. Figure 1**. According to the QC analysis, we also calculated the calibrated ccfDNA concentrations (based on the percentage of concentrations at 150–250 bps) in a large subgroup of 55 out of 67 samples (including 40 patients with ACA and 15 HS). We observed an excellent correlation between the total and calibrated concentrations (*R* = 0.993, *P* < 0.001, **Suppl. Figure 2**); we therefore used total concentrations for the present study.

Total ccfDNA concentrations were considered “positive” or “high” when above our cut-off of 0.146 ng/µL, previously calculated as mean + 2SD in a cohort of 25 HS using the same pipeline [16].

### ccfDNA sequencing for identification of somatic mutations

 Sequencing was carried out on ccfDNA samples isolated from patients with ACA before adrenalectomy. In brief, sequencing libraries were prepared using a customised gene panel, i.e. Cell3™ Target Custom NGS Panel (Nonacus), which included 32 genes known to be associated with or suspected to be involved in adrenocortical tumours (**Suppl. Table 1**). Specifically, the panel included cAMP/PKA-related genes (*GNAS*,* PRKACA*,* PRKAR1A*,* PDE8B)* - frequently mutated in CPA, *CTNNB1* - mostly altered in MACS and NFAT, as well as *KCNJ5* and *CACNA1D* - frequently mutated in APA, together with a number of genes frequently mutated in adrenocortical carcinoma [16]. The protocol consisted of End-repair and A-tailing of ccfDNA followed by ligation of Illumina UMI adapters, bead-based purification, PCR amplification, a second bead-based purification and library quality check. Equimolar amount of each library was pooled for an overnight hybridisation with custom design NGS panel. After probe capture on Streptavidin beads and subsequent washes, NGS library pool enriched for regions of interest was again PCR amplified. Captured pool was sequenced on NextSeq2000 sequencer using paired-end sequencing with NextSeq 2000 P3 Reagents (200 Cycles) kit (Illumina, San Diego CA, US). Due to the bi-institutional nature of our study, sequencing data and subsequent variant calling from seven of the ccfDNA samples (ACA2, ACA4, ACA9, ACA11, ACA20, ACA25 and ACA26) was done using the procedure outlined previously [16].

## Tumour tissue DNA isolation and sequencing

Matched formalin-fixed paraffin-embedded (FFPE) tumour tissue was available in patients who underwent adrenalectomy. Tumour localization was annotated by an expert pathologist and tumour cell content was assessed in a representative FFPE slide by haematoxylin-eosin staining before DNA isolation. Tumour cell content reached a high fraction (median 90%, range 60–95). DNA was isolated from tumour material using the Omega Bio-Tek Mag-Bind^®^ FFPE DNA Kit (Omega Bio-Tek, Georgia, USA).

Library preparation of tumour DNA (T-DNA) and paired normal DNA was conducted with the Cell3™ Target Library preparation kit (with fragmentation) (Nonacus, Birmingham, UK). The protocol consisted of preliminary DNA fragmentation followed by End-repair and A-tailing of fragmented DNA. The remaining protocol was the same as for ccfDNA sequencing (see above).

## Somatic variant calling

Raw sequencing reads were aligned to the GRCh38 human reference genome and candidate somatic variants were called using TNhyplotyper2 (Sentieon, City, Country) following the pipeline recommended by the Broad Institute for Somatic short variant discovery (SNVs and Indels) on tumour samples with paired normal DNA to filter out germline variants. One patient (ACA2) did not have a paired normal sample and, in this case, variants were called running the protocol in tumour-only mode using a panel of normals to filter out germline variants instead. Samples from ccfDNA underwent unique molecular identifier (UMI) extraction and consensus calling in place of duplicate marking as described in Bieler et al. [21] to capture variants at extremely low variant allele frequencies (VAFs). An additional orientation bias model (Sentieon) was used to remove strand bias or slippage artefacts and variants with a total read depth less than 10 in either T-DNA /ccfDNA or normal samples were excluded from consideration. Variant candidates were then rejected based on a further set of stringent filters: T-DNA (respectively ccfDNA) variants were retained only if their estimated lower VAF limit was at least 0.05 (respectively 0.015) provided their estimated upper VAF limit in paired normal DNA remained less than 0.05, based on a 95% confidence interval calculated using the Clopper-Pearson method for the binomial distribution. To control for the effect of stringent filtering on variant calling in ccfDNA samples, we re-evaluated the variants called in the absence of this stringent filter and inspected read alignments manually using Interactive Genomics Viewer. None of the ccfDNA variants called with the relaxed filter were found in paired T-DNA samples, confirming that the absence of biologically relevant variants called on ccfDNA is not an artifact of a stringent filtering criterion. Variants were classified by their germline pathogenicity as reported in the ClinVar database (REF: PMID 24234437 and 36063163).

### Statistical analysis

Statistical analyses were performed using GraphPad Prism version 9 (GraphPad Software). Continuous data are shown as median (interquartile range (IQR)). Categorical variables are expressed as numbers and percentages. The comparison of non-parametric continuous data was performed by using the Mann–Whitney test or Kruskal– Wallis test followed by Dunn’s post hoc test. Categorical variables were compared by χ2 test or Fisher’s test, as appropriate. The relationship between continuous variables was determined by linear regression (Pearson’s correlation coefficient if the 2 variables were normally distributed or Spearman’s correlation coefficient for not normally distributed variables). P value less than 0.05 was considered statistically significant.

## Results

### Patient characteristics

We included 44 patients with ACA (29 women; median age 55.3 years, range 21–83): 17 CPA-MACS, 9 CPA-CS, 12 APA, and 6 NFAT. The control group included 23 HS: 14 women; median age 35 years (range 23–62). An overview of the demographics and clinical characteristics of patients with ACA at the time of diagnosis is shown in Table 1. Patients with ACA had similar sex distribution compared to HS. Patients with NFAT and CPA-MACS were significantly older than HS, while APA and CPA-CS cases had a similar age distribution. The proportion of unilateral/bilateral adrenal masses was similar among the ACA subgroups. APA represented the smallest tumours (Table 1). As expected, CPA-CS had the highest post-ONDST cortisol levels and the lowest DHEAS levels (Table 1).

**Table 1:** Demographic data, clinical parameters and circulating cell-free DNA (ccfDNA) levels in the entire study cohort `

	HS (*n* = 23)	ACA Cohort				
		Overall cohort (*n* = 44)	NFAT (*n* = 6)	APA (*n* = 12)	CPA-MACS (*n* = 17)	CPA-CS (*n* = 9)
Women, n (%)	14 (60.87)	29 (65.91)	2 (33.33)	6 (50.00)	13 (76.47)	8 (88.89)
		NS	NS	NS	*p* = 0.057^b^	***p*** **= 0.025**^**b**^ *p* = 0.061^c^
Age (years), median (IQR)	34.50 (29.75–44.50)	56.50 (45.00–68.25)	66.50 (55.50–73.75)	44.50 (36.25–53.75)	65.00 (56–71.50)	46.00 (29.50–58)
		***p*** **< 0.001**^**a**^	***p*** **= 0.002**^**a**^	NS	***p*** **< 0.001** ^**a**^ ***p*** **= 0.019**^**c**^	***p*** ** = 0.025** ^**d**^
Unilateral tumours, n (%)	NA	36 (81.82)	6 (100)	11 (91.67)	12 (70.59)	7 (77.78)
				***p*** **= 0.002**^**b**^	NS	NS
Maximum tumour diameter (cm)^#^, median (IQR)	NA	2.80 (2.05–3.73)	3.25 (2.35–4.73)	1.4 (1.23–2.42)	3 (2.65–4.15)	2.8 (2.20–3.60)
				*p* = 0.055^b^	***p*** **= 0.002**^**c**^	NS
Serum cortisol in the 1 mg DST, (nmol/L), median (IQR)	NA	85 (31.50–275.0)	39.50 (54.00-28.99)	28 (25.00–28.00)	101.0 (76.00–245.5)	518.0 (281.0–650.0)
				NS	***p*** **= 0.005**^**c**^	***p*** **< 0.001**^**b**^ ***p*** **< 0.001**^**c**^
Missing information, n		3	0	3	0	0
Serum DHEAS, (µmol/L), median (IQR)	NA	1.08 (0.48–2.11)	1.26 (0.52–3.24)	2.85 (1.52–7.59)	1.08 (0.40–2.31)	0.49 (0.34–0.93)
				NS	*p* = 0.095^c^	***p*** **= 0.005**^**c**^
Missing information, n		5	0	5	0	0
Plasma ACTH, (ng/L), median (IQR)	NA	6.00 (4.00–16.50)	11.80 (6.25–39.40)	18.00 (10.70–27.10)	5.20 (4.00–13.00)	5 (3.00–16.50)
				NS	NS	NS
Missing information, n		11	2	7	2	0
Plasma Total ccfDNA concentrations, (ng/µL), median (IQR)	0.05 (0.00–0.08)	0.12 (0.05–0.19)	0.12 (0.09–0.19)	0.09 (0.04–0.14)	0.12 (0.06–0.18)	0.18 (0.05–0.47)
		***p*** **< 0.001**^**a**^	NS	NS	*p* = 0.053^a^	***p*** ** = 0.005** ^**a**^

Categorical variables are reported as n (%); statistical comparison performed by χ2 test or Fisher’s test. Continuous variables are reported as median (InterQuartile Range = IQR); statistical analyses performed by the Kruskal–Wallis test followed by Dunn’s post hoc test

HS, healthy subject; ACA, adrenocortical adenoma; NA, not applicable; NFAT, non-functioning adrenal tumour; APA, aldosterone-producing adenoma; CPA-CS, cortisol-producing adenoma with overt Cushing’s syndrome; CPA-MACS, cortisol-producing adenoma with mild autonomous cortisol secretion; 1 mg DST, 1 mg overnight dexamethasone suppression test; DHEAS, dehydroepiandrosterone sulfate; ACTH, adrenocorticotropin hormone

NS, non significant (vs. all groups). Only p values < 0.10 are reported for simplicity, p values < 0.05 in bold

^a^p-value vs. HS; ^b^p-value vs. NFAT; ^c^p-value vs. APA; ^d^p-value vs.CPA-MACS

^#^ For bilateral tumours, the maximum diameter of the larger adrenal mass was considered

A total of 17/44 patients underwent adrenalectomy for removal of the primary tumour in presence of clinically-relevant steroid excess: 7 CPA-CS, 5 CPA-MACS, and 5 APA.

### ccfDNA concentrations and clinical parameters

**Fig. 1:**
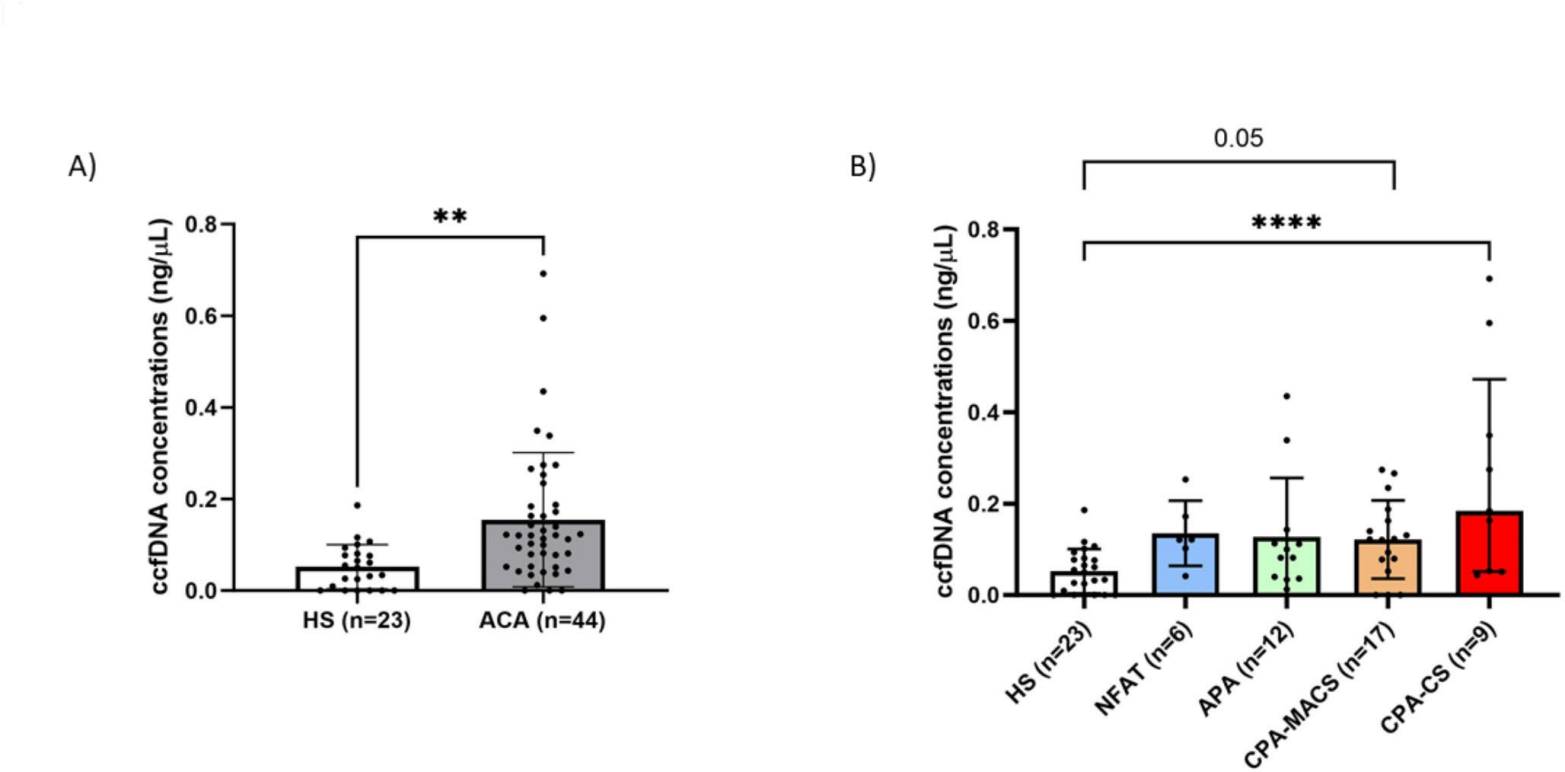
Circulating cell-free DNA (ccfDNA) concentrations in patients with adrenocortical adenomas (ACA). Panel (**A**) Comparison of ccfDNA concentrations between 23 healthy subjects (HS) and 44 patients with ACA. Statistics by Mann–Whitney test. **p < 0.05. Panel (**B**) Comparison of ccfDNA concentrations between HS and ACA subgroups: non-functioning adrenal tumour (NFAT), aldosterone-producing adenomas (APA), cortisol-producing adenoma with mild autonomous cortisol secretion (CPA-MACS) and cortisol-producing adenoma with overt Cushing’s syndrome (CPA-CS). Statistics by Kruskal–Wallis test followed by Dunn’s post hoc test. **p < 0.05, ****p < 0.001

Patients with ACA had significantly higher total ccfDNA concentrations than HS: median: 0.12 (IQR 0.05–0.19) vs. 0.05 (IQR 0.00–0.08) ng/µL (*P* < 0.001, Fig. 1A; Table 1). Considering our cut-off of 0.146 ng/µL, 64% of ACAs presented undetectable or low ccfDNA levels (negative). Total ccfDNA concentrations did not correlate with neither age nor sex, neither in the entire cohort nor when considering HS and ACA separately (**Suppl. Figure 3**). The high ccfDNA levels were mostly driven by patients with CPA-CS [median 0.18 ng/µl (IQR 0.05–0.47), *P* = 0.005 vs. HS by Kruskall-Wallis test, Fig. 1B; Table 1] or CPA-MACS (median 0.12 ng/µl (IQR 0.06–0.18), *P* = 0.053 vs. HS), while concentrations in NFAT and APA did not differ significantly from HS.

**Fig. 2:**
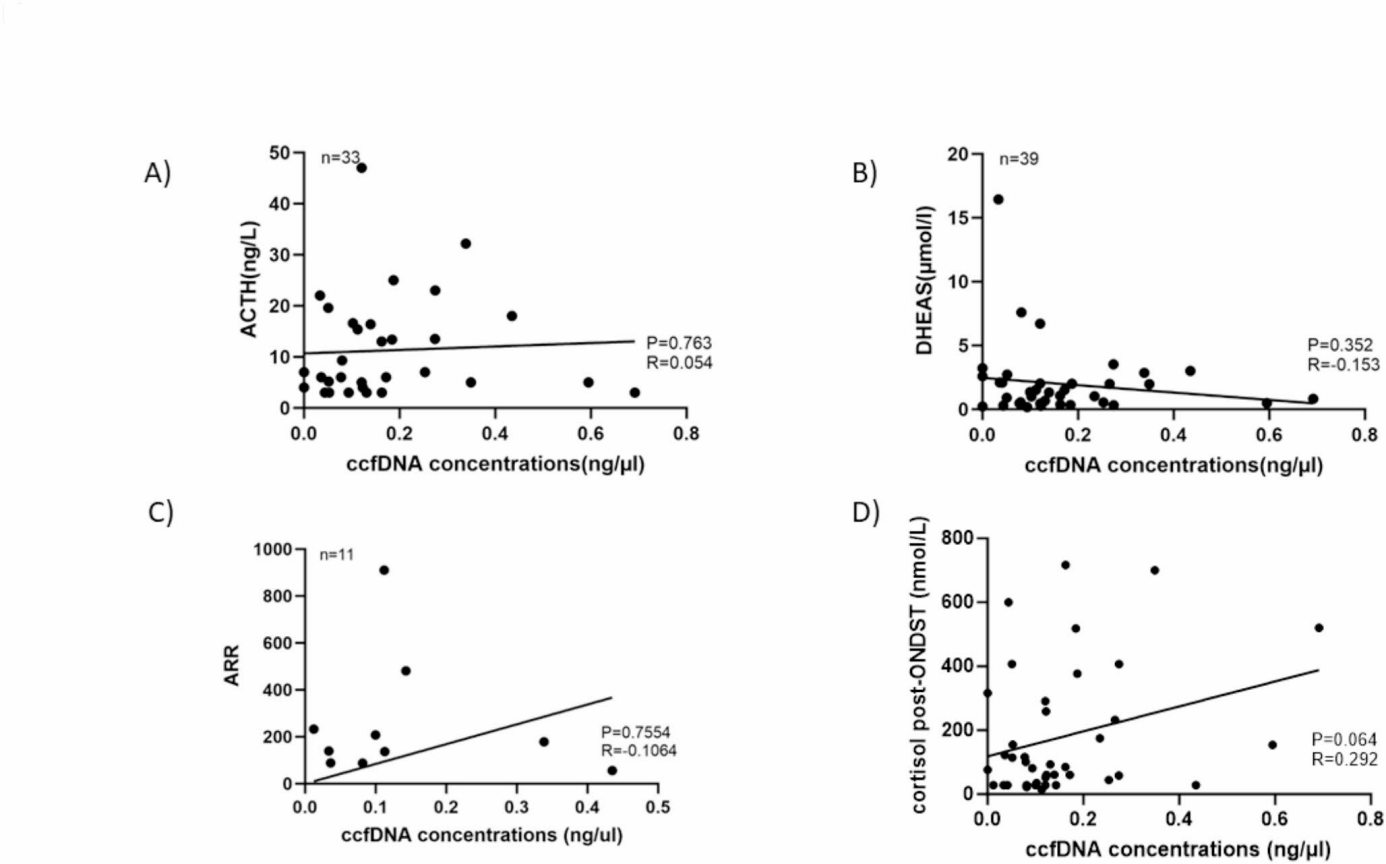
Correlation between circulating cell-free DNA (ccfDNA) concentrations and different hormonal levels in patients with adrenocortical adenomas (ACA). (**A**) Correlation between adrenocorticotropin hormone (ACTH) and ccfDNA concentrations (*n* = 33). (**B**) Correlation between dehydroepiandrosterone sulphate (DHEAS) and ccfDNA concentrations (*n* = 39). (**C**) Correlation between aldosterone-renin-ratio (ARR) and ccfDNA concentrations in 11 patients with aldosterone-producing adenomas (APA) (*n* = 11). (D) Correlation between cortisol after 1 mg DST and ccfDNA concentrations (*n* = 41). P values were determined with Spearman’s correlation coefficient

ccfDNA concentrations did not correlate significantly with age, tumour diameter, ACTH, DHEAS, or ARR levels (Fig. 2A-C), but we observed a mild trend toward a positive correlation with cortisol levels after ONDST when considering all patients (*n* = 44, *R* = 0.292, *P* = 0.064, Fig. 2D).

### Somatic variants at T-DNA and ccfDNA level

We performed targeted sequencing of 17 T-DNA samples (7 CPA-CS, 5 CPA-MACS, and 5 APA). We detected 9 different somatic variants in 4 known ACA-driver genes in 9/17 samples (53%) (Table 2). As expected, most altered genes belonged to the cAMP signalling pathway for CPA-CS and Wnt/β-catenin pathway for CPA-MACS and Potassium channels for APA. In particular, two pathogenetic/likely pathogenic hot spot *PRKACA* variants were observed in two CPA-CS, one missense (p.Leu206Arg) and one in-frame insertion (p.Leu199_Cys200insTrp). Moreover, four T-DNA samples carried alterations in exon 3 of *CTNNB1*: a missense mutation of unknown clinical significance in one CPA-CS (p.Leu46Pro), a pathogenic/likely pathogenic missense mutation in one CPA-MACS (p.Ser45Pro), and an in-frame deletion (pThr42_Pro44del) followed by a pathogenic/likely pathogenic missense mutation (pSer45Phe) on the same allele in another CPA-MACS, and a pathogenic/likely pathogenic missense mutation (pSer45Phe) in one CPA-MACS. Two pathogenic/likely pathogenic *KCNJ5* missense mutations (p.Gly151Arg, p.Leu168Arg) and one missense mutation of unknown clinical significance (p.Ile1015Thr) were found in three APA (Table 2).

**Table 2:** Somatic variants detected by next-generation sequencing in tumour-DNA samples of 17 patients with adrenocortical adenomas who underwent adrenalectomy (7 CPA-CS, 5 CPA-MACS, and 5 APA)

Patient ID	Diagnosis	Gene name	Chromosome	Variant classification	Variant type	HGVSc	HGVSp	Clinical significance
ACA4	CPA-CS	*CTNNB1*	chr3	Missense	SNP	c.137T > C	p.Leu46Pro	Unknown
ACA26	CPA-CS	*PRKACA*	chr19	Missense	SNP	c.617T > G	p.Leu206Arg	Likely pathogenic/pathogenic
ACA29	CPA-CS	*PRKACA*	chr19	In_Frame_Ins	INS	c.597_599dup	p.Leu199_Cys200insTrp	Pathogenic
ACA24	CPA-MACS	*CTNNB1*	chr3	Missense	SNP	c.133T > C	p.Ser45Pro	Likely pathogenic/pathogenic
ACA100	CPA-MACS	*CTNNB1*	chr3	In_Frame_Del	DEL	c.124_132del	p.Thr42_Pro44del	Unknown
ACA100	CPA-MACS	*CTNNB1*	chr3	Missense	SNP	c.134 C > T	p.Ser45Phe	Pathogenic/likely pathogenic
ACA115	CPA-MACS	*CTNNB1*	chr3	Missense	SNP	c.134 C > T	p.Ser45Phe	Pathogenic/likely pathogenic
ACA6	APA	*KCNJ5*	chr11	Missense	SNP	c.451G > A	p.Gly151Arg	Pathogenic/likely pathogenic
ACA30	APA	*KCNJ5*	chr11	Missense	SNP	c.503T > G	p.Leu168Arg	Likely pathogenic
ACA32	APA	*CACNA1D*	chr3	Missense	SNP	c.3044T > C	p.Ile1015Thr	Unknown

Abbreviations: CPA-CS, cortisol-producing adenoma with overt Cushing’s syndrome; CPA-MACS, cortisol-producing adenoma with mild autonomous cortisol secretion; APA, aldosterone-producing adenomas; HGVS, Human Genome Variation Society; HGVSc, HGVS coding sequence; HGVSp, HGVS protein sequence; SNP (Single Nucleotide Polymorphism); INS (Insertion); DEL (Deletion)

We did not detect any pathogenic or likely pathogenic somatic variants in known ACA-driver genes in any of the paired 17 ccfDNA samples from these patients. This corresponded to 9/17 positive T-DNA (53%) vs. 0/17 positive ccfDNA (*P* = 0.0009 by Fisher’s exact test).

Of the 12 variants called in 17 ccfDNA samples, six mapped to small, entirely novel deletions in the *GNAS* gene from two patients (one CS-CPA and one APA). The remaining six variants mapped to novel SNVs within *MEN1*, *MSH2*, *EGFR* and *KMT2D*, none of which have previously been reported mutated in ACA. Importantly, none of these SNVs or *GNAS* variants were found in paired T-DNA samples. No ccfDNA variants could therefore confidently be ascribed to somatic mutations in circulating DNA originating from the adrenal tumour.

## Discussion

In the present study, we performed the first, to our knowledge, systematic ccfDNA analysis in a well-defined cohort of patients with benign adrenocortical adenomas.

Genetic alterations in the cAMP/PKA pathway have been observed in up to 50–60% of unilateral CPA associated with overt CS and 10–15% of unilateral MACS cases [9], correlating with the severity of hormone excess and clinical outcomes in CPA-CS [20, 22, 23]. Previous studies suggested that a small percentage of patients with MACS may progress to CS [24], highlighting the importance of monitoring and management of patients with MACS. Moreover, somatic variants in *KCNJ5* are found in ~ 40% of unilateral APA, which typically correlate with a more severe phenotype but also better clinical outcomes after adrenalectomy [25]. However, the use of molecular profiling in the clinical management of patients with ACA currently represents an underutilized opportunity to refine disease monitoring and treatment, since the detection of somatic mutations on tumour tissue is only possible after adrenalectomy. We therefore hypothesized that somatic variants detected in the ccfDNA of patients with ACA could provide pre-operative evidence of specific genetic signatures to inform clinical decisions and management strategies. To test this hypothesis, we performed a comprehensive ccfDNA analysis in prospectively collected samples from 44 patients with different types of ACA.

Considering total ccfDNA concentrations, we found that ccfDNA levels were overall significantly higher in patients with ACA compared to HS. This difference was mainly driven by the presence of more elevated ccfDNA concentrations specifically in CPA-CS and CPA-MACS subgroups. In literature, few data are available about ccfDNA levels in benign tumours, but, overall, they have been reported as relatively low [17–19]. In a recent study, we investigated ccfDNA concentrations in patients with adrenocortical carcinoma: we observed that 96% of ACC compared to 36% of ACA presented total ccfDNA concentrations above our HS cut-off [16].

ccfDNA concentrations did not correlate with any of the evaluated demographic, clinical or hormonal parameters. The relationship between ccfDNA levels and post-ONDST cortisol levels showed a trend to a positive correlation. This trend, even if not significant, together with the maximum concentrations observed in plasma of patients with CPA-CS and CPA-MACS, suggests that the higher concentrations observed in ACA compared to HS is mainly driven by the impact of excessive circulating cortisol, rather than to the nature of the tumour. This is further supported by the notion that cfDNA seems to be predominantly released by cells of the hematopoietic lineage and glucocorticoids are known to affect leukocyte profiles (with higher cortisol values associated with a greater number of neutrophils) [26].

Taking advantage of our previously established pipeline for detecting somatic variants in the ccfDNA from patients with adrenocortical carcinoma (ACC) [16], we tested whether somatic variants could also be detected in blood samples collected from 17 patients with ACA before adrenalectomy. We performed targeted sequencing in matched T-DNA/ccfDNA samples using a highly sensitive customized panel including ACC/ACA-specific driver genes. Within T-DNA, somatic variants were identified in 53% of ACA patients with a frequency distribution in line with previous studies (i.e., *PRKACA* in 30% of CPA-CS, *CTNNB1* in 50% of CPA-MACS and 15% of CPA-CS, *KCNJ5* in 50% of APA and *CACNA1D* in 9% of APA) [9–13, 27–29]. Of note, we also found a somatic *CTNNB1* variant of unknown clinical significance in one CPA-CS and a somatic *CACNA1D* variant of unknown clinical significance in one APA.

By comparison with T-DNA, we did not detect any known ACA-driver mutations within the ccfDNA of patients who underwent adrenalectomy. While we did call some novel *GNAS* variants in ccfDNA from two of the 17 patients, such deletions are not typical of hotspot *GNAS* activating mutations known to be associated with ACA. Importantly, these and the handful of other variants called in ccfDNA were not identified in T-DNA from any of the patients within our study. Although it cannot be entirely ruled out that these originate from DNA secreted into circulation from unsampled regions of tumours, we hypothesise that such calls are technical artefacts of library preparation and/or sequencing errors that have persisted beyond our data processing and filtering efforts designed to capture somatic variants with extremely low VAFs. Alternative explanations for the paucity of ACA driver events detected in ccfDNA could include inadequate sample size, exceptionally low ccfDNA concentrations obtained from patients ACA as compared to levels reported in patients with solid cancers [30], or current limitations on the depth of next-generation sequencing techniques.

This study has some limitations. The first one is the relatively limited sample size when looking at subgroups with different steroid secretion or with matched tumour and ccfDNA samples that could potentially affect statistical power. Another limitation is represented by the choice of a customized, adrenal tumour-specific gene panel. This targeted approach allows for the precise identification of specific genetic alterations pivotal for adrenal tumorigenesis. However, given the relatively limited research in this area, some variants might have been missed.

In conclusion, despite finding ACA-specific somatic alterations in half of tumours from our cohort, we could not identify any corresponding somatic mutations within the ccfDNA of these patients. Therefore, this approach seems ineffective for detecting pathogenic variants using currently available technology in patients with ACA before surgery.

## Electronic supplementary material

Below is the link to the electronic supplementary material.

Supplementary Material 1

Supplementary Material 2

Supplementary Material 3
