# Supplementary material for: ccfDNA analysis for the classification of adrenocortical adenomas
Source: J Endocrinol Invest. 2025 Feb 1;48(5):1207–16. doi: 10.1007/s40618-025-02540-5 (PMC12049379; doi:10.1007/s40618-025-02540-5)
Supplement: Supplementary file 1 — Supplementary Material 1 [file 40618_2025_2540_MOESM1_ESM.docx]

**Suppl Table 1. List of the 32 genes included in the customized panel for targeted next-generation sequencing.**

|  | **Gene Name** | **Pathway or family** | **ACA or ACC specific** |
| --- | --- | --- | --- |
| 1 | *CDK4* | Rb/p53 | ACC |
| 2 | *TP53* | Rb/p53 | ACC |
| 3 | *CDKN2A* | Rb/p53 | ACC |
| 4 | *RB1* | Rb/p53 | ACC |
| 5 | *MDM2* | Rb/p53 | ACC |
| 6 | *CTNNB1* | Wnt/beta catenin pathway | ACA and ACC |
| 7 | *APC* | Wnt/beta catenin pathway | ACA and ACC |
| 8 | *ZNRF3* | Wnt/beta catenin pathway | ACC |
| 9 | *DAXX* | Chromatin remodelling | ACC |
| 10 | *TERT* | Chromatin remodelling | ACC |
| 11 | *MEN1* | Chromatin remodelling | ACA and ACC |
| 12 | *ATRX* | Chromatin remodelling | ACC |
| 13 | *KDM6A* | Chromatin remodelling | ACC |
| 14 | *KMT2D* | Chromatin remodelling | ACC |
| 15 | *MSH2* | Mismatch DNA repair | ACC |
| 16 | *MLH1* | Mismatch DNA repair | ACC |
| 17 | *GNAS* | cAMP/PKA pathway | ACA and ACC |
| 18 | *PRKAR1A* | cAMP/PKA pathway | ACA and ACC |
| 19 | *ATM* | Homologous DNA Repair | ACC |
| 20 | *BRCA2* | Homologous DNA Repair | ACC |
| 21 | *FGFR3* | Angiogenic Factors | ACC |
| 22 | *KDR* | Angiogenic Factors | ACC |
| 23 | *EGFR* | Angiogenic Factors | ACC |
| 24 | *NF1* | Others | ACC |
| 25 | *NOTCH1* | Others | ACC |
| 26 | *PTCH1* | Others | ACC |
| 27 | *SETD2* | Others | ACC |
| 28 | *PRKACA* | cAMP/PKA pathway | ACA (CPA) |
| 29 | *PDE8B* | cAMP/PKA pathway | ACA (CPA) |
| 30 | *PDE11A* | cAMP/PKA pathway | ACA (CPA) |
| 30 | *ARMC5* | Others | ACA (PBMAH) |
| 31 | *KCNJ5* | Potassium channels | ACA (APA) |
| 32 | *CACNA1D* | Potassium channels | ACA (APA) |

**Abbreviations:** ACA, adrenocortical adenoma; ACC, adrenocortical carcinoma; CPA, cortisol-producing adenoma; PBMAH, primary bilateral macronodular adrenal hyperplasia; APA, aldosterone-producing adenoma.
