# Supplementary material for: ccfDNA analysis for the classification of adrenocortical adenomas
Source: J Endocrinol Invest. 2025 Feb 1;48(5):1207–16. doi: 10.1007/s40618-025-02540-5 (PMC12049379; doi:10.1007/s40618-025-02540-5)
Supplement: Supplementary file 2 — Supplementary Material 2 [file 40618_2025_2540_MOESM2_ESM.docx]

**Legend to the Supplementary Figures**

**Supplementary Figure 1. Representative examples of quality control (QC) by TapeStation (D1000 ScreenTape®) in two samples from adrenocortical adenoma (ACA) and two healthy subjects (HS) samples.** Reports of Sample Integrity were automatically generated by the TapeStation Analysis Software 4.1.1

A) Cortisol-producing adenoma with overt Cushing Syndrome (CPA-CS, ACA26); B) Cortisol-producing adenoma with mild autonomous cortisol secretion (CPA-MACS, ACA100); C) HS7; D) HS9.

**Supplementary Figure 2. Correlation between total ccfDNA concentrations and calibrated ccfDNA concentrations in adrenocortical adenoma (ACA) and healthy subjects (HS).**

A) Correlation between total ccfDNA concentrations and calibrated ccfDNA concentrations in entire group. P values were determined with Spearman’s correlation coefficient. B) Correlation between total ccfDNA concentrations and calibrated ccfDNA concentrations in health subjects (HS). C) Correlation between total ccfDNA concentrations and calibrated ccfDNA concentrations in patients with ACA.

**Supplementary Figure 3. ccfDNA concentrations in patients with HS and adrenocortical adenomas divided by sex.**

A) ccfDNA concentrations in males and females with health subjects (HS). Data are shown as median and interquartile range, and the upper and the lower whiskers represent, respectively, the 90 and the 10 percentiles.

B) ccfDNA concentrations in males and females with adrenocortical adenomas (ACA).

C) Correlation between ccfDNA concentrations and ages in HS.

D) Correlation between ccfDNA concentrations and ages in patients with ACA. P values were determined with Spearman’s correlation coefficient.
