# Supplementary material for: ccfDNA analysis for the classification of adrenocortical adenomas
Source: J Endocrinol Invest. 2025 Feb 1;48(5):1207–16. doi: 10.1007/s40618-025-02540-5 (PMC12049379; doi:10.1007/s40618-025-02540-5)
Supplement: Supplementary file 3 — Supplementary Material 3 [file 40618_2025_2540_MOESM3_ESM.pptx]

## Slide 1
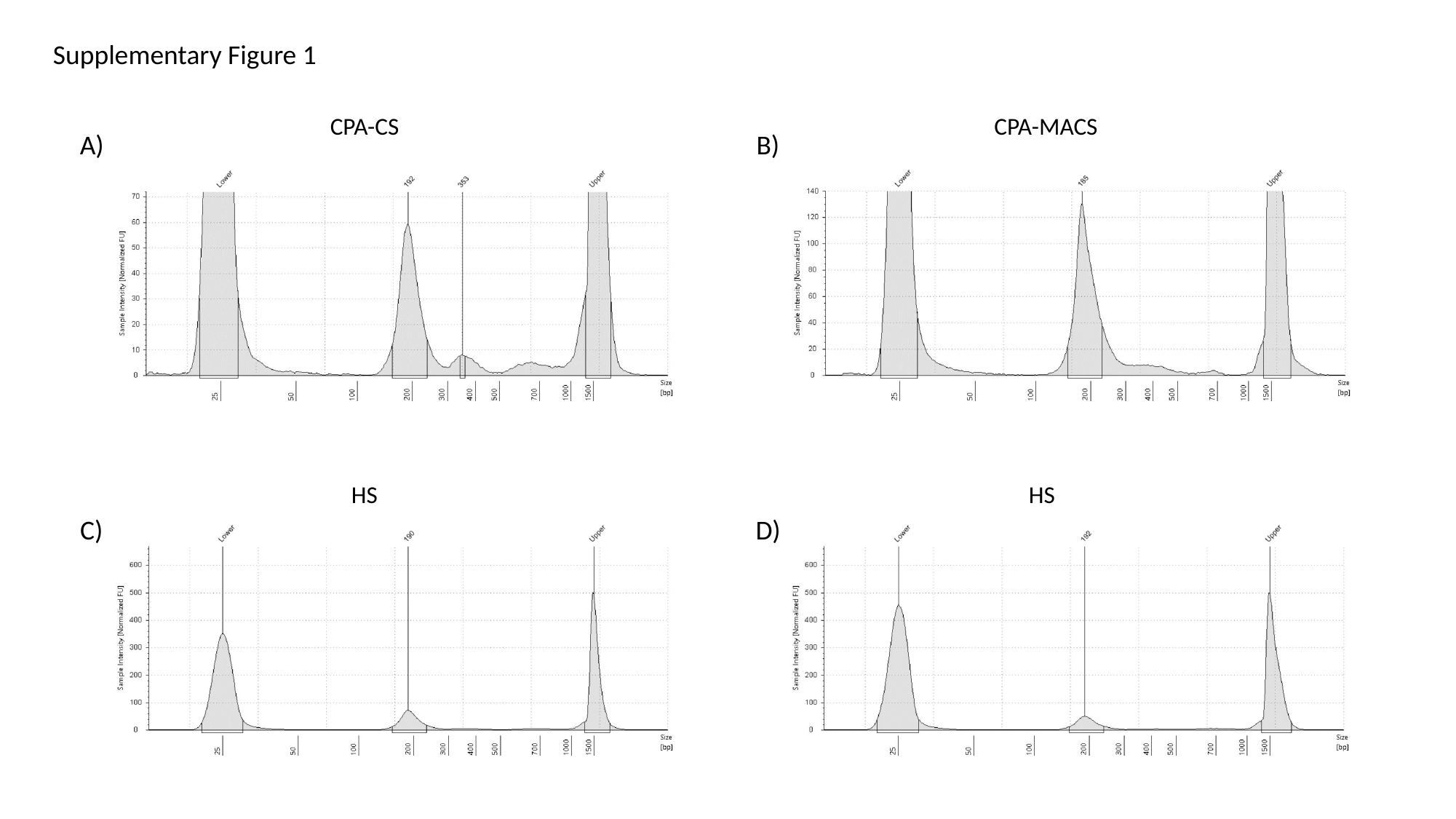

Supplementary Figure 1
CPA-CS
CPA-MACS
B)
A)
HS
HS
C)
D)

## Slide 2
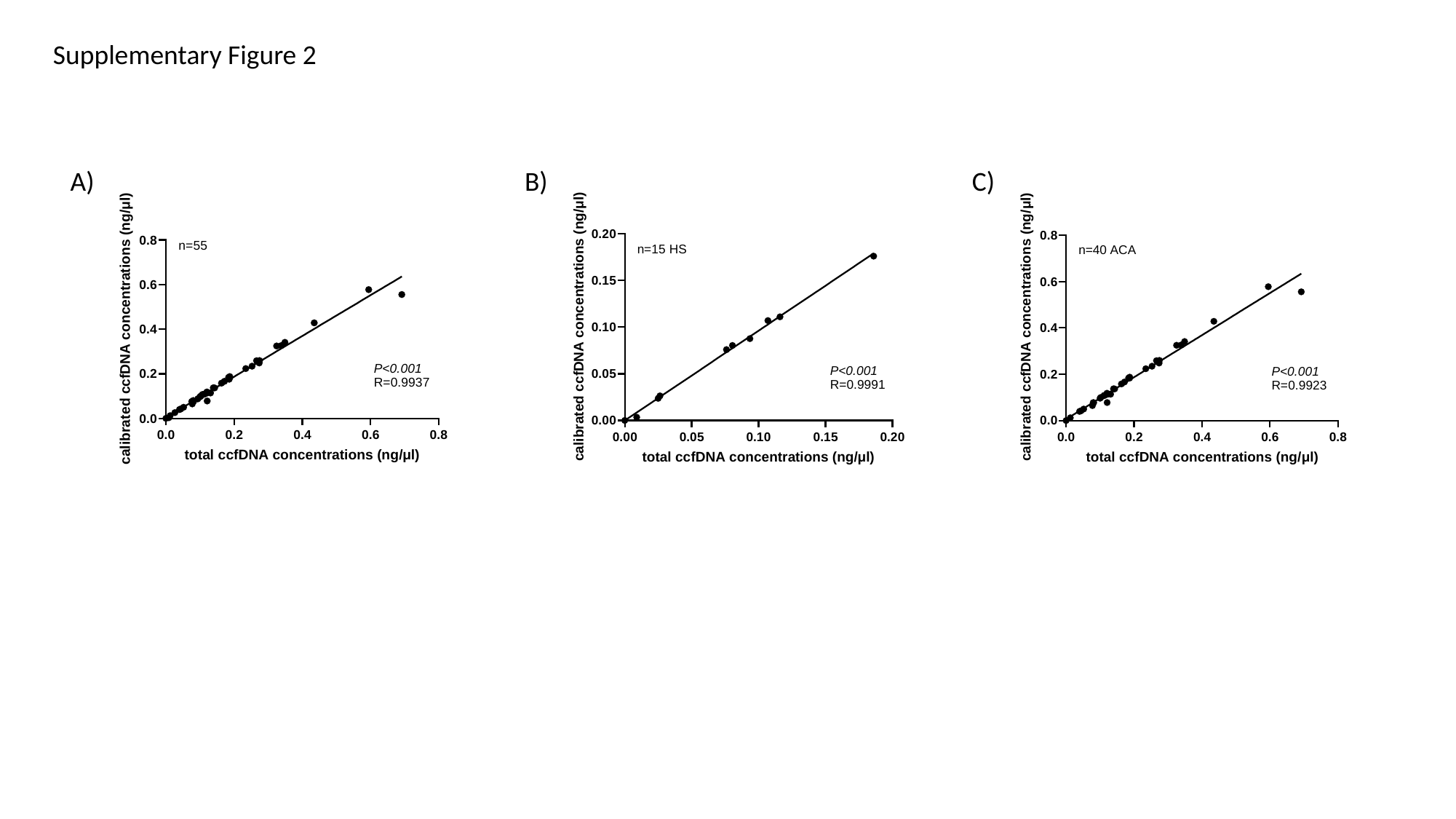

Supplementary Figure 2
B)
C)
A)

## Slide 3
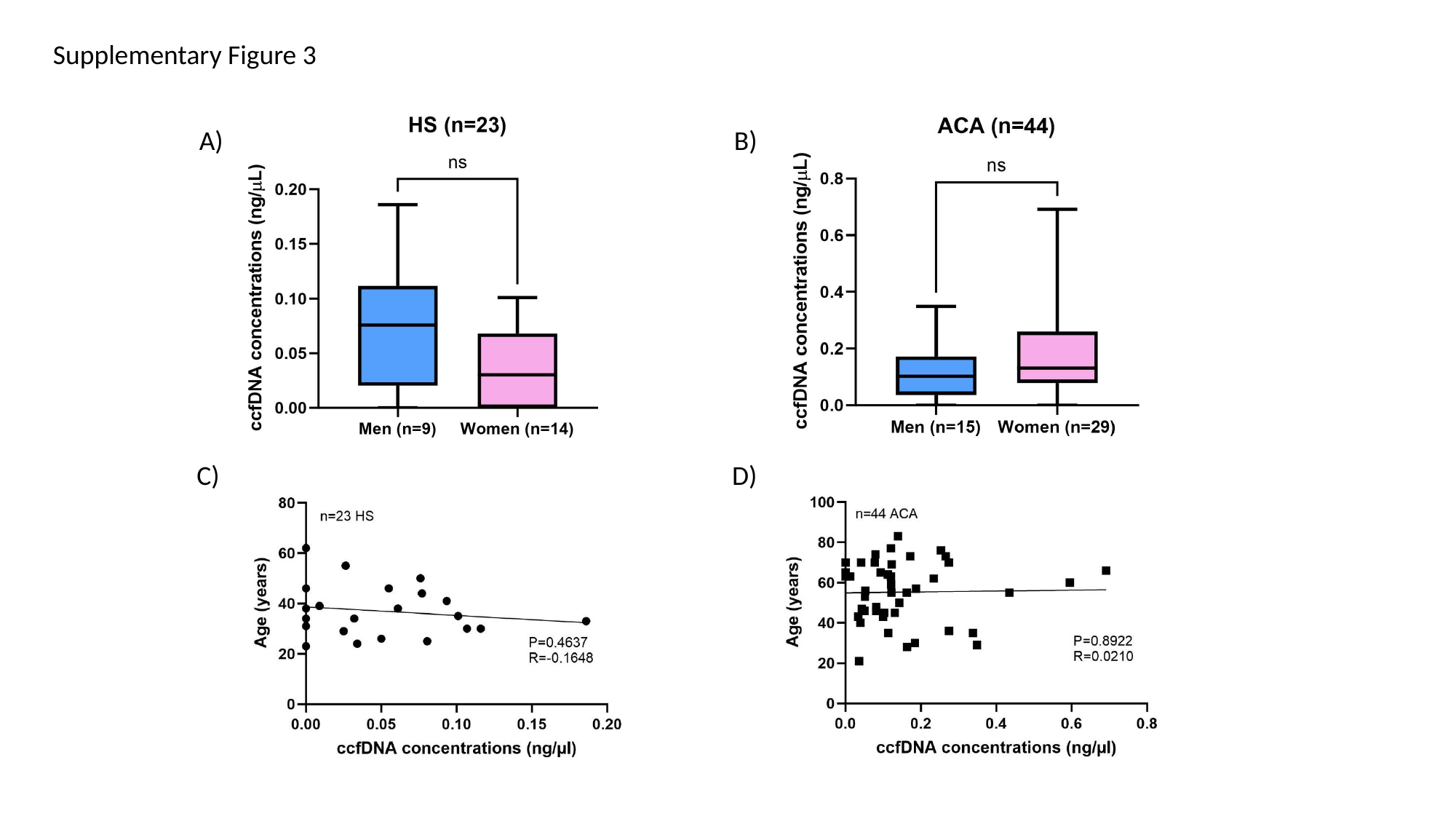

Supplementary Figure 3
A)
B)
C)
D)
